# Identification and Analysis of Resistance to Northern Corn Leaf Blight in Maize Germplasm Resources

**DOI:** 10.3390/plants14203171

**Published:** 2025-10-15

**Authors:** Bing Meng, Junwei Yang, Lixiu Tong, Qingli Liu, Dongfeng Zhang, Wen-Xue Li, Jianjun Wang, Yunbi Xu, Zifeng Guo, Canxing Duan

**Affiliations:** 1 State Key Laboratory of Crop Gene Resources and Breeding, National Engineering Laboratory for Crop Molecular Breeding, Institute of Crop Sciences, Chinese Academy of Agricultural Sciences, Beijing 100081, Chinaliwenxue@caas.cn (W.-X.L.);; 2 Institute of Maize Research, Shanxi Agricultural University, National Agricultural Experimental Station for Plant Protection in Xinzhou, Xinzhou 034000, China; 3 State Key Laboratory of Crop Germplasm Innovation and Molecular Breeding, Syngenta Biotechnology (China) Co., Ltd., Beijing 102206, China; 4 Seeds Research, Syngenta Crop Protection, LLC, Research Triangle Park, Durham, NC 27709, USA

**Keywords:** NCLB, resistance source, heterotic group, 123N, *Exserohilum turcicum*

## Abstract

Northern corn leaf blight (NCLB), caused by the fungus *Exserohilum turcicum*, is one of the most significant foliar diseases in maize worldwide, with its severity being highly influenced by environmental conditions. An effective strategy used to control NCLB involves screening diverse maize germplasm for resistant sources through multi-environment inoculation assays, ultimately aiming to develop resistant varieties. This study systematically evaluated 711 maize germplasm accessions with rich genetic diversity. The evaluation was conducted under four location–year environment combinations (Shangluo, Shaanxi Province, China in 2014–2015 and Xinzhou, Shanxi Province, China in 2021–2022) using artificial inoculation with physiological race 123N (or races 1, 2, 3, N). The results showed that the estimated variances of genotype, environment, and genotype-by-environment interaction were all highly significant (*p* < 0.01). Significant correlations (*p* < 0.01) were observed among replicates within each environment, with correlation coefficients (*r*) ranging from 0.67 to 0.88. At the Xinzhou trial in 2021, four replicates were inoculated with four physiological races (1, 2, 3, and N), revealing highly significant correlations (*r* = 0.77–0.80, *p* < 0.01) among them. The disease severity of the tropical germplasm was significantly lower (*p* < 0.001) than that of the temperate germplasm. Among the temperate subgroups, the PA and PB (groups A and B germplasms derived from modern US hybrids) subgroups exhibited lower disease severity, with the PB subgroup showing the lowest, while the Iodent and Reid subgroups exhibited higher susceptibility. The disease severity responses to the four physiological races were highly positively correlated (*r* = 0.77–0.80, *p* < 0.001), and their correlations with the composite inoculation (race 123N) ranged from 0.65 to 0.83. Based on the resistance evaluations across four location–year environment combinations, the 711 maize accessions were classified into five categories: 20 were highly resistant, 236 resistant, 205 moderately resistant, 237 susceptible, and 13 highly susceptible. The findings indicate that the tropical germplasm and the temperate PB subgroup are major sources of NCLB resistance.

## 1. Introduction

Northern corn leaf blight (NCLB), caused by the fungus *Exserohilum turcicum*, is one of the major foliar diseases of maize. It occurs widely in maize-growing regions worldwide, with reports of its incidence and management documented across Asia [1], Europe [2], Africa [3], and South America [4]. In addition to infecting maize leaves, *E. turcicum* can also colonize husks and leaf sheaths, though the most typical symptoms appear on leaves as spindle-shaped lesions. The pathogen typically first infects the lower leaves and gradually progresses upward, potentially spreading to the entire plant under severe conditions [5]. In high-humidity environments, grayish-black mold may develop on the lesion’s surface, primarily on the abaxial side of the leaves, impairing photosynthesis and nutrient accumulation. This leads to shriveled or deteriorated kernels, ultimately reducing yield [6]. NCLB is an airborne disease favored by moderate temperatures and high humidity. Typically, it can cause yield losses of 15%–20%, while infections occurring before silking may result in losses as high as 40%–50% [7]. To respond to NCLB, the most economical, reliable, and environmentally friendly method is to identify highly resistant inbred lines to develop resistant varieties.

Maize resistance to NCLB can be classified into qualitative (monogenic) resistance and quantitative (polygenic) resistance. Qualitative resistance is conferred by *Ht* genes. To date, 11 resistance genes (*Ht1*, *Ht2*, *Ht3*, *HtM*, *HtP*, *HtNB*, *Htn1*, *NNc*, *St*, *ht4*, and *rt*) have been identified. However, *Ht2* and *Ht3* were later confirmed to be identical at the genomic sequence level, representing a novel allelic variant of *Htn1*. *Htn1*/*Ht2*/*Ht3* encodes a wall-associated receptor-like kinase [8,9]. The *Ht1* gene has also been cloned and encodes a nucleotide-binding, leucine-rich repeat immune receptor [10]. Nevertheless, qualitative resistance is often unstable, particularly in tropical regions, where it may fail due to emerging virulent races or climate sensitivity. Quantitative resistance is broad-spectrum and highly heritable. Through linkage and association analysis, multiple quantitative trait loci (QTL) have been mapped [11,12,13]. While most of these QTL explain only a small proportion of the phenotypic variation [14], some QTL contribute to larger genetic effects [15].

Based on virulence against maize lines carrying different *Ht* genes, *E. turcicum* can be classified into distinct physiological races [16]. In China, 16 races (0, 1, 2, 3, N, 12, 13, 1N, 23, 2N, 3N, 123, 12N, 23N, 13N, and 123N) have been identified, with race 0 being the most prevalent, followed by race 1. The 123N race, also termed the super race, can overcome all four major *Ht* genes (*Ht1*, *Ht2*, *Ht3*, and *HtN*) [17]. Besides China, this virulent race has been reported in multiple regions worldwide [18,19]. In China during the 1960s, NCLB caused severe damage only in limited regions. Following the widespread deployment of hybrid varieties, the balance between the pathogen and the host was disrupted, resulting in progressive annual increases in NCLB severity. By the 1980s, the deployment of the NCLB-resistant inbred line Mo17 enabled the development of resistant hybrids that effectively contained large-scale epidemics. However, by the late 1980s, pathogen evolution had generated new physiological races capable of infecting Mo17—which carries the *Ht* resistance gene—thereby intensifying disease pressure. This culminated in a 1993 NCLB outbreak across multiple maize-growing regions, causing substantial yield losses. To address persistent challenges, resistance identification against diverse physiological races, particularly those with heightened virulence, remains imperative [20].

Maize utilizes heterosis, and inbred lines can be classified into different heterotic groups based on combining ability or molecular markers. In U.S. maize breeding, two major groups (Stiff Stalk, SS; Non-Stiff Stalk, NSS) dominate. International Maize and Wheat Improvement Center (CIMMYT) lines are divided into Tropical Heterotic Group A and Tropical Heterotic Group B [21,22]. Chinese maize inbreds are typically classified into eight heterotic groups, including Tang Sipingtou (SPT), derived from Chinese landraces [23]. Different heterotic groups not only contribute to hybrid performance but also exhibit varying responses to biotic and abiotic stresses [24,25]. The tropical germplasm generally shows stronger disease resistance and stress tolerance compared with the temperate germplasm [26]. Among temperate subgroups, the PB heterotic group, derived from U.S. hybrid germplasm including accessions 78599, 78641, 78698, and 87001, demonstrates robust resistance to multiple diseases, representing a key genetic resource for disease resistance breeding [27,28]. Heterotic groups exhibit distinct characteristics due to divergent geographical origins and primary cultivation ranges. The identification of heterotic groups with high NCLB resistance would represent a significant advancement in breeding NCLB-resistant cultivars. This would enable breeders to strategically exploit established combining ability patterns between heterotic groups, facilitating the efficient development of highly resistant hybrids for NCLB management.

When conducting this study, we had three primary objectives: (1) to identify stable resistance sources with low environmental sensitivity by evaluating a large panel of maize germplasm for NCLB resistance across multiple environments; (2) to determine the primary sources of NCLB resistance by analyzing resistance variation among heterotic groups and providing insights into NCLB-resistant breeding; and (3) to assess the virulence variation and correlations among different physiological races (1, 2, 3, N, and 123N) through inoculations on the same germplasm set, thereby selecting accessions with broad-spectrum resistance.

## 2. Results and Analysis

### 2.1. Analysis of Individual Environments

Significant variations in NCLB severity were observed across the four location–year environment combinations. The most severe infection occurred in Shangluo2014, with a mean resistance rating of 7.01 across all accessions, which was classified as susceptible. In contrast, Xinzhou2022 showed the mildest infection, with a mean resistance rating of 4.10, indicating a moderate resistance level that approaches the resistant category. Across all four location–year environment combinations, the overall mean resistance rating of the 711 accessions was 5.12, corresponding to a moderately resistant level (Table 1). Analysis of variance (ANOVA) revealed that genotypic variance was highly significant (*p* < 0.001) for all of the materials tested across the four environments, with the highest genotypic variance observed in Shangluo2015 (5.03 ***). The genotype-by-environment interaction variance also reached highly significant levels. The broad-sense heritability (*H*^2^) estimates for NCLB resistance were 0.93, 0.85, 0.95, and 0.93 for Shangluo2014, Shangluo2015, Xinzhou2021, and Xinzhou2022, respectively, with a combined heritability of 0.94 (Table 1).

**Figure 1:**
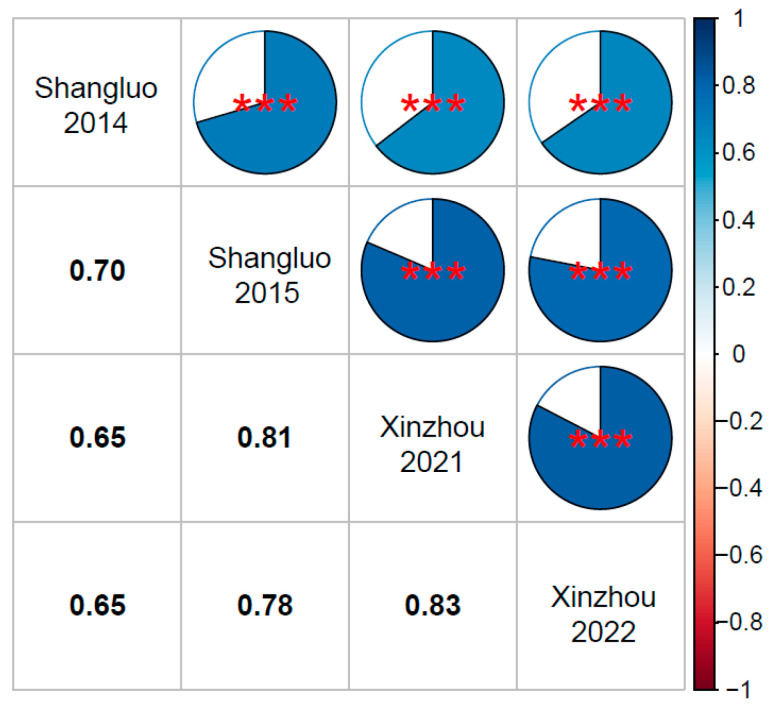
Correlation analysis of northern corn leaf blight severity among replicates for maize inbred lines across four location–year environment combinations. *** denotes *p* < 0.001.

Correlation analysis demonstrated highly significant (*p* < 0.001) associations between replicates within each environment. The correlation coefficients between two replicates were 0.88 and 0.75 for Shangluo2014 and Shangluo2015, respectively (Supplementary Table S1). Among eight replicates in Xinzhou2021, the correlation coefficients ranged from 0.67 to 0.79 (Supplementary Table S1), while those among the four replicates in Xinzhou2022 varied between 0.74 and 0.78 (Supplementary Table S1). The phenotypic correlations across the four location–year environment combinations were all highly significant (*p* < 0.001), with coefficients (*r*) ranging from 0.65 to 0.83 (Figure 1). Notably, despite using different physiological races (1, 2, 3, and N) for inoculation in Xinzhou2021, significant correlations (*p* < 0.001) were observed with Shangluo2014 (*r* = 0.65 ***), Shangluo2015 (*r* = 0.81 ***), and Xinzhou2022 (*r* = 0.83 ***) (Figure 1).

### 2.2. Resistance Variation Among Heterotic Groups

**Figure 2:**
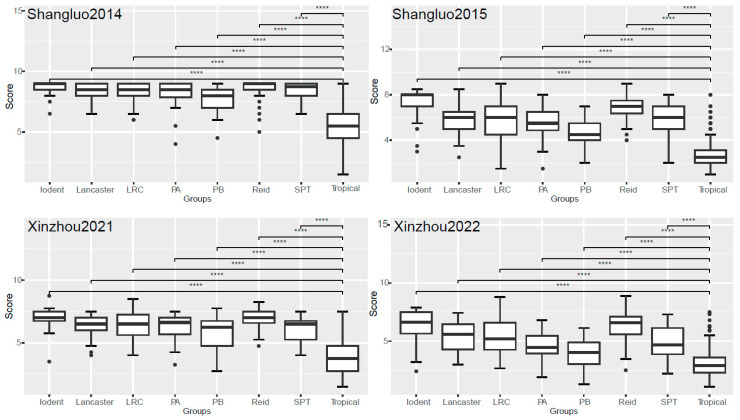
Disease severity ratings of eight maize heterotic groups across different location–year environment combinations. LRC, Lvda Red Cob; PA and PB, groups A and B germplasms derived from modern US hybrids; SPT, derivatives from the Tangshan Sipingtou Chinese landrace. **** denotes *p* < 0.001.

Based on the classification by Guo et al. [29], the 711 maize inbred lines were categorized into eight heterotic groups commonly utilized in Chinese breeding programs. The evaluation of NCLB resistance across all four location–year environment combinations (Shangluo2014, Shangluo2015, Xinzhou2021, and Xinzhou2022) revealed that the tropical germplasm exhibited significantly lower disease severity (*p* < 0.001) compared with other groups, with mean resistance ratings of 5.39, 2.79, 3.98, and 3.03, respectively (Figure 2). Among temperate groups, the PB group (group B germplasm derived from modern US hybrids) demonstrated the highest resistance, showing consistently lower disease severity compared with the other temperate groups across all the environments (mean ratings: 7.69, 4.76, 5.60, and 3.95). In contrast, the Iodent and Reid groups exhibited higher susceptibility, with Iodent being the most vulnerable (Figure 2; Table 2). Comparative analysis across the environments indicated that disease severity was more pronounced in Shangluo2014 and Xinzhou2021, with minimal variation among the temperate groups. In Shangluo2015 and Xinzhou2022, distinct resistance hierarchies emerged, consistently ranking as follows: tropical > PB > PA (group A germplasm derived from modern US hybrids) > SPT (derivatives from the Tangshan Sipingtou Chinese landrace) > LRC (Lvda Red Cob) > Lancaster > Reid > Iodent. The SPT group displayed intermediate resistance, with mean severity ratings of 8.46, 6.09, 6.03, and 5.04 across the four environments (Figure 2; Table 2).

Further, we conducted an analysis of variance (ANOVA) regarding NCLB resistance among the heterotic groups in the two environments (Xinzhou2021 and Xinzhou2022), where complete material planting was achieved (Table 2). The results demonstrated that both genotypic variance and genotype-by-environment interaction variance reached highly significant levels (*p* < 0.001) across all eight groups in both location–year environment combinations. Specifically, in Xinzhou2021, the tropical and PB groups exhibited larger genetic variances, while in Xinzhou2022, the LRC and Iodent groups showed greater genetic variances. All eight heterotic groups maintained high broad-sense heritability (*H*^2^) values. The tropical group displayed the highest *H*^2^ (0.93) in Xinzhou2021, whereas the Iodent and Reid groups showed the peak *H*^2^ values (0.90) in Xinzhou2022.

### 2.3. Resistance Induced by Different Physiological Races

**Figure 3:**
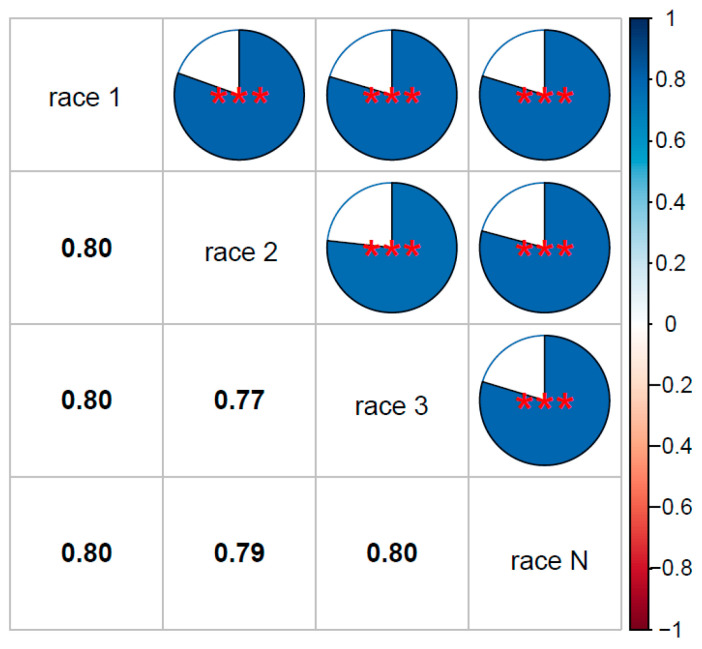
Correlation analysis of disease severity following inoculation with four physiological races (1, 2, 3, and N). *** denotes *p* < 0.001.

In Xinzhou2021, four different physiological races (1, 2, 3, and N) were used for inoculation, with two replicates for each race. The results showed that the correlation between two replicates was highest for NCLB infection caused by race 3, while the correlation was lowest for race 1 infection. The correlation coefficients of disease severity between two replicates for races 1, 2, 3, and N were 0.690, 0.722, 0.767, and 0.713, respectively, with all correlations between replicates reaching highly significant levels (*p* < 0.001) (Figure 3). Significant correlations (*p* < 0.001) were observed among all four physiological races (1, 2, 3, and N), with phenotypic correlation coefficients (*r*) ranging from 0.77 to 0.80 for disease severity under inoculation with these four races (Figure 3). Combined with the correlations between Xinzhou2021 and the other three location–year environment combinations (Figure 1), these results indicate that the pathogenicity of the four physiological races showed little difference from that of 123N.

**Figure 4:**
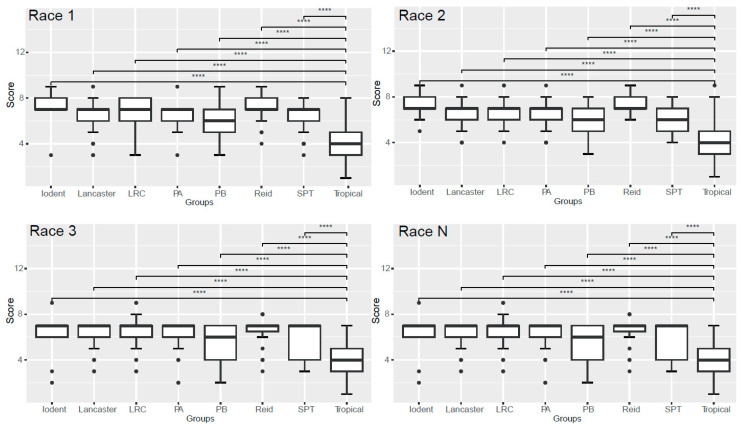
Disease severity statistics for eight heterotic groups after separate inoculation with physiological races 1, 2, 3, and N. LRC, Lvda Red Cob; PA and PB, groups A and B germplasms derived from modern US hybrids; SPT, derivatives from the Tangshan Sipingtou Chinese landrace. **** denotes *p* < 0.001.

Furthermore, using data from the 2021 Xinzhou trial site, we compared the resistance of different heterotic groups to physiological races (Figure 4). The results showed that, similar to inoculation with the 123N race, when inoculated separately with races 1, 2, 3, and N, both the tropical and PB groups showed lower average disease severity. The disease severity of the tropical group was significantly lower (*p* < 0.001) than that of the other groups, and also significantly lower than that of the PB group, with mean values of 4.20, 4.16, 3.86, and 3.70, respectively. Similarly, the Iodent and Reid groups showed higher disease severity, with the disease severity values for the Iodent group being 7.28, 7.41, 6.56, and 6.72 (Figure 4).

### 2.4. Comprehensive Analysis of Resistance Evaluation Results Across Four Environments

**Figure 5:**
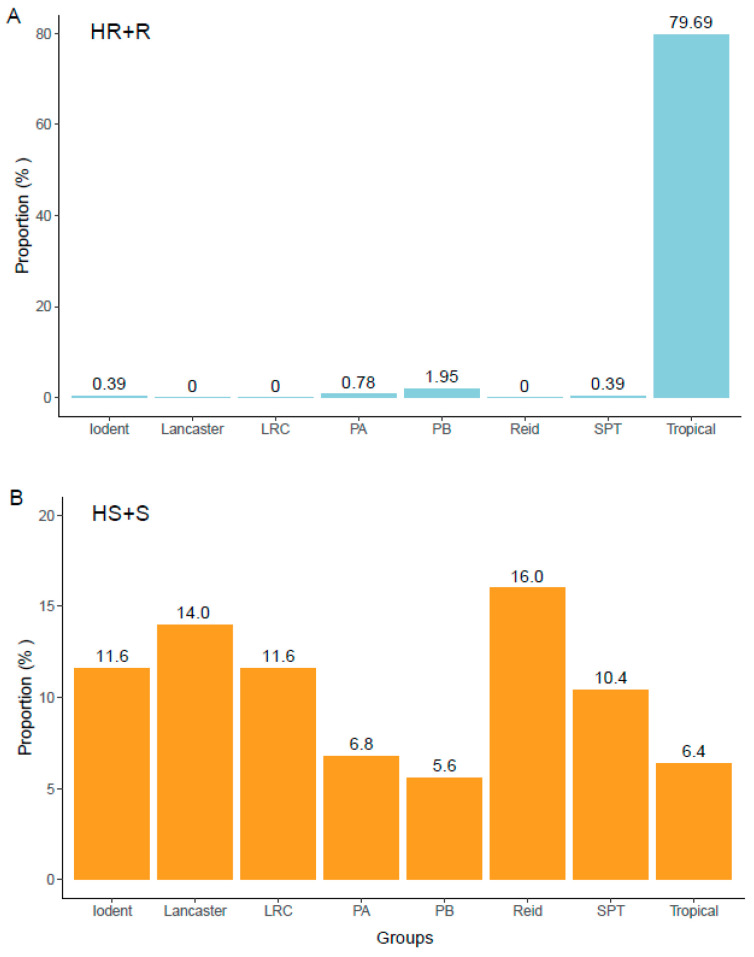
Distribution patterns of accessions rated highly resistant (HR) and resistant (R) (**A**) or susceptible (S) and highly susceptible (HS) (**B**) across four location–year environment combinations among heterotic groups. LRC, Lvda Red Cob; PA and PB, groups A and B germplasms derived from modern US hybrids; SPT, derivatives from the Tangshan Sipingtou Chinese landrace.

Based on the resistance evaluation results from four location–year environment combinations, among the 711 maize materials, 20 were identified as highly resistant (HR), 236 as resistant (R), 205 as moderately resistant (MR), 237 as susceptible (S), and 13 as highly susceptible (HS). Further classification according to their groups revealed that among the 20 highly resistant accessions, 15 were from the tropical group (Supplementary Table S2). Among the 13 highly susceptible germplasms, 5 were from the Iodent group, 5 were from the Reid group, 2 were from the LRC group, and 1 was from the other groups. Of the 256 materials identified as resistant or higher (HR+R), the tropical group had the largest number (204 materials) (Figure 5A). Among the 250 materials identified as susceptible or highly susceptible, the Reid group had the highest number (40 materials) (Figure 5B).

Taking the complete material planting results from Xinzhou2021 and Xinzhou2022 as examples for analysis, the results showed (Table 3) that the tropical group included 19 materials identified as highly resistant, while the other groups had no materials rated as highly resistant. The Iodent, LRC, and Reid groups had one, one, and four materials evaluated as highly susceptible, respectively, while other groups had no materials rated as highly susceptible. The most resistant material came from the tropical group with a resistance rating score of 1.48. The least resistant material came from the LRC group with a resistance rating score of 8.65.

## 3. Discussion

Northern corn leaf blight (NCLB) frequently occurs in spring maize regions of Northern China and cool mountainous areas of southwest maize production zones, with the development of the disease being significantly influenced by environmental conditions [30]. In this study, using consistent inoculation methods and spore concentrations, there were substantial differences in the overall NCLB severity across the four location–year environment combinations. Shangluo2014 showed the highest average severity (7.01), with both environmental variance and genotype-by-environment interaction variance reaching highly significant levels. This indicates that environmental conditions significantly affect NCLB’s development, and an evaluation of NCLB resistance in maize germplasm should be conducted through multi-environment testing. The genotypic variance reached highly significant levels across all four location–year environment combinations, demonstrating significant differences in NCLB resistance among the 711 tested accessions. Different resistant germplasm resources can be selected from these materials. The high heritability indicates that NCLB resistance is a genetically controlled trait that can be addressed through breeding resistant varieties [3].

The genetics of NCLB resistance involves not only major genes but also polygenic resistance [31]. NCLB-associated QTL intervals and significantly associated SNPs have been identified at multiple loci, including bin1.06–1.07, bin6.05 [32], and bin3.04 [12]. The cloned lesion mimic-related gene *Mexicana lesion mimic 1* (*ZmMM1*) promotes the accumulation of the ZmMM1 protein, which suppresses the transcription of *ZmMT3*, a downstream gene that negatively regulates plant immune responses, thereby enhancing resistance to NCLB [33]. Existing research shows that polygenic resistance to NCLB is primarily additive, with minimal nonadditive effects [34,35]. This suggests that the resistance of inbred lines is closely related to hybrid resistance, and when breeding resistant hybrids, resistant inbreds should be selected at first [36].

Maize inbreds can be classified into heterotic groups based on their combining ability [37]. The analysis of resistance across heterotic groups indicates that the tropical germplasm and PB germplasm show stronger resistance, suggesting that the tropical germplasm is an excellent material for NCLB-resistant breeding and gene mining, while temperate breeding should focus on PB germplasm [38]. It is noteworthy that the tropical group, which, in this study, was represented by a substantially larger number of accessions, exhibited highly significant genotypic variance, indicating considerable variation in NCLB resistance among these accessions. Since the Iodent germplasm showed high susceptibility across all four location–year environment combinations, it should be used cautiously in high-NCLB-incidence regions of China, though it could serve as a susceptible parent material for genetic mapping.

Different physiological races of NCLB cause varying symptoms [17,18,39,40,41,42,43]. The results from Xinzhou2021 showed high correlations among the disease phenotypes caused by different races. In other words, if a material shows susceptibility when inoculated with race 1, it will also likely show susceptibility when inoculated with races 2, 3, or N. This suggests two possibilities: firstly, the four dominant genes *Ht1*, *Ht2*, *Ht3*, and *HtN* may be closely linked; secondly, most NCLB resistance genes are quantitative trait loci, as quantitative resistance typically provides broad-spectrum resistance without showing race-specific phenotypes [8,44,45,46]. The analysis of heterotic groups also showed consistent phenotypic responses to inoculation with races 1, 2, 3, and N, confirming the validity of using race 123N for evaluation instead of individual race inoculations.

Through comprehensive analysis across four location–year environment combinations, this study identified several highly resistant materials, though most were from tropical groups. How to modify the photoperiod/thermal sensitivity of the tropical germplasm for use in Chinese NCLB-resistant breeding programs remains an ongoing and urgent problem [47,48,49,50]. The adaptability of tropical maize germplasm can be improved through gradually cultivating it from low-latitude to high-latitude regions [51]. Additionally, PB germplasm containing tropical ancestry represents an important resistance source [52,53,54]. Among the highly susceptible materials identified, besides the Iodent group, LRC materials showed poor resistance. Many hybrids planted in Northeast China contain LRC ancestry, partly explaining why Northeast China is one of the high-NCLB-incidence regions [55,56]. Future breeding efforts should focus on improving NCLB resistance in LRC germplasm [57,58].

## 4. Materials and Methods

### 4.1. Plant Materials

A total of 711 maize accessions were used for the NCLB resistance evaluation, including 402 temperate materials provided by the Institute of Crop Sciences, Chinese Academy of Agricultural Sciences (CAAS), and 309 tropical accessions from the CIMMYT. Based on genotypic data obtained from 40K mSNP liquid chip assays, genetic diversity analysis and principal component analysis (PCA) were performed [29]. The temperate materials comprised seven major heterotic groups used in Chinese maize breeding: Iodent (32 accessions), Lancaster (49), Reid (43), LRC (derivatives from the Lvda red coda Chinese landrace) (47), SPT (derivatives from the Tangshan Sipingtou Chinese landrace) (39), PA (28), and PB (39) (groups A and B germplasms derived from modern US hybrids, respectively) [29]. The remaining 125 inbred lines did not fall into the heterotic groups predominantly used in Chinese maize breeding programs (Supplementary Table S2).

### 4.2. Field Experimental Design and Management

Field trials were conducted in four environments: Shangluo, Shaanxi Province, China (33.87° N, 109.94° E) in 2014 and 2015 (Shangluo2014 and Shangluo2015); and Xinzhou, Shanxi Province, China (38.47° N, 112.95° E) in 2021 and 2022 (Xinzhou2021 and Xinzhou2022). These two trial sites, Shangluo and Xinzhou, were selected because their environmental conditions are generally conducive to the development of NCLB in maize (Supplementary Table S3). The planting dates were 5 May and 6 May for Shangluo2014 and 2015, and 14 May and 18 May for Xinzhou2021 and 2022, respectively. The experimental design followed an incomplete randomized block design with single-row plots (3 m length, 13 plants per row, 0.25 m plant spacing, and 0.6 m row spacing). The replication numbers were 2 replicates (2014–2015), 8 replicates (2021), and 4 replicates (2022). Standard local agronomic practices were followed for fertilization and field management (Supplementary Table S1).

### 4.3. Fungal Isolates and Culture

To evaluate the resistance of 711 maize accessions against *Exserohilim turcicum* physiological races, races 1, 2, 3, and N were inoculated at the Xinzhou trial site in 2021. In the other trial environments, the highly virulent race 123N was used to ensure stringent selection pressure for durable resistance (Supplementary Table S1). The 123N race for inoculations was provided by CAAS (for the 2014 and 2015 inoculations) and Syngenta China (for the 2022 inoculation). In Xinzhou2021, four distinct races (1, 2, 3, and N) provided by CAAS were inoculated (two replicates per race). Fungal cultures were initially grown on PDA medium; PDA plates colonized by the fungal strain were aseptically cut into segments and transferred to sterile Erlenmeyer flasks containing autoclaved sorghum grains. The flasks were incubated within a 12 h photoperiod at 20–25 °C for 7–14 days, with daily agitation to ensure uniform mycelial colonization of all grain surfaces. Subsequently, the colonized grains were spread onto porcelain trays sterilized with 75% ethanol and covered with sterile newspaper moistened with minimal sterile water to maintain humidity. Sporulation was induced by incubation in complete darkness at ambient room temperature.

### 4.4. Inoculation and Disease Assessment

In all four location–year environment combinations, inoculation was performed when 50% of the plants had fully expanded 12 of their leaves. After microscopic confirmation of abundant spore production, spores were washed from sorghum grains and adjusted to a concentration of 1 × 10^6^ spores/mL. The suspension was mixed with 0.05% Tween-20 and applied using a sprayer during cool within humid evening conditions. Additionally, the washed sorghum grains were evenly distributed into the leaf whorls of the maize plants to ensure successful inoculation.

Disease evaluation was conducted 30 days after natural pollen shedding, following the established protocols [59,60]. Each plant within rows was manually rated using a 9-grade scale based on lesion area coverage (Table 4) [61]: Grade 1: high resistance; Grade 9: high susceptibility.

The mean resistance score (${\mathrm{NCLBS}}_{M}$) for each accession was calculated as follows:

$${\mathrm{NCLBS}}_{M}=\frac{\sum_{i}^{n}NCLBS}{n}$$

where *NCLBS* = northern corn leaf blight score per plant (1–9 scale); ${\mathrm{NCLBS}}_{M}$ = mean northern corn leaf blight score; and *i* = 1 (first plant) to *n* (last plant in row). The final resistance classifications followed the scale outlined in Table 4.

### 4.5. Data Analysis

For data processing, Microsoft Excel and R (psych package for correlation analysis; ggplot2 for visualization) were used [62,63,64]. ANOVA and heritability calculations were performed using QTL IciMapping 4.2 [65] with the linear model, as follows:

$$y_{ijk}=\mu +g_{i}+e_{j}+b_{k/j}+{ge}_{ij}+\varepsilon_{ijk}$$

where i = genotype, j = environment, k = replicate, $y_{ijk}$ = phenotypic value, *μ* = grand mean, $g_{i}$ = genotype effect, $e_{i}$ = environment effect, $b_{k/j}$ = block effect within environment, ${ge}_{ij}$ = G×E interaction, and $\varepsilon_{ijk}$ = residual error.

The broad-sense heritability (H^2^) was calculated as follows [66]:

Single environment:

$$H^{2}=\sigma_{G}^{2}/(\sigma_{G}^{2}+\sigma_{\varepsilon }^{2}/r)$$

Multiple environments:

$$H^{2}=\sigma_{G}^{2}/(\sigma_{G}^{2}+\sigma_{GE}^{2}/l+\sigma_{\varepsilon }^{2}/lr)$$

where $\sigma_{G}^{2}$ represents the genetic variance, $\sigma_{\varepsilon }^{2}$ represents the residual error variance, $\sigma_{GE}^{2}$ represents genotype × environment interaction variance, r is the number of replications in each environment, and l is the number of environments.

The 2021 dataset comprising four races (1, 2, 3, and N) was processed using the following two approaches: Firstly, without distinguishing between physiological races, the Xinzhou2021 data were treated as eight replicates and analyzed collectively with other location–year environment combinations. Secondly, we separately analyzed the Xinzhou2021 data to examine resistance differences in northern corn leaf blight caused by different physiological races.

## 5. Conclusions

In this study, the multi-environment evaluation of 711 accessions demonstrated that NCLB severity is strongly influenced by environmental conditions, making multi-location testing necessary. Genetic factors predominantly control resistance (*H*^2^ = 0.85–0.95), enabling the selection of resistant breeding materials. The tropical germplasm and temperate PB group showed superior resistance, while the Iodent and Reid groups were the most susceptible. High correlations (*r* = 0.77–0.80) among race-specific responses suggest polygenic, broad-spectrum resistance mechanisms. The identified resistant accessions provide valuable genetic resources for NCLB-resistant breeding programs.

## Figures and Tables

**Table 1:** Variance components for 711 maize inbred lines across four location–year environment combinations.

Environments	Number ^※^	NCLB Score		Variance Components				*H* ^2^
		Mean ± SD	Range	σ G 2	σ ε 2	σ E 2	σ G E 2	
Shangluo2014	556	7.01 ± 1.94	1.50–9.00	4.21 ***	0.48			0.93
Shangluo2015	540	4.51 ± 2.28	1.00–9.00	5.03 ***	1.34			0.85
Xinzhou2021	711	5.13 ± 1.99	1.50–8.75	2.79 ***	1.14			0.95
Xinzhou2022	711	4.10 ± 1.75	1.08–8.90	2.79 ***	0.87			0.93
Total	711	5.12 ± 2.14	1.00–9.00	2.68 ***	1.04	0.84 ***	0.41 ***	0.94

Note: $\sigma_{G}^{2}$, $\sigma_{E}^{2}$, $\sigma_{GE}^{2}$, and $\sigma_{\varepsilon }^{2}$ represent genotypic, environment, genotype-by-environment interaction, and error variance, respectively; *H*^2^ represents heritability; *** indicates significance at *p* < 0.001. ^※^ The actual number of surveyed lines was reduced in the Shangluo trial due to severe lodging.

**Table 2:** Analysis of variance (ANOVA) of NCLB ratings among heterotic groups at the Xinzhou site (2021 and 2022).

Heterotic Group	Number	NCLB Score (Mean ± SD)		σ G 2		σ G E 2	*H* ^2^	
		2021	2022	2021	2022		2021	2022
Iodent	32	6.99 ± 0.82	6.33 ± 1.36	0.59 ***	1.79 ***	0.55 ***	0.86	0.90
Lancaster	49	6.35 ± 0.83	5.31 ± 1.23	0.58 ***	1.32 ***	0.38 ***	0.83	0.87
LRC	47	6.38 ± 1.05	5.32 ± 1.50	0.97 ***	2.08 ***	0.28 ***	0.87	0.92
PA	28	6.21 ± 1.00	4.62 ± 1.16	0.90 ***	0.91 ***	0.25 ***	0.87	0.73
PB	39	5.60 ± 1.31	3.95 ± 1.15	1.64 ***	0.97 ***	0.30 ***	0.93	0.82
Reid	43	6.93 ± 0.72	6.38 ± 1.31	0.42 ***	1.60 ***	0.36 ***	0.79	0.90
SPT	39	6.03 ± 1.08	5.04 ± 1.25	1.05 ***	1.34 ***	0.14 **	0.89	0.86
Tropical	309	3.98 ± 1.44	3.03 ± 1.16	1.92 ***	1.08 ***	0.37 ***	0.93	0.83

Note: LRC, Lvda Red Cob; PA and PB, groups A and B germplasms derived from modern US hybrids; SPT, derivatives from the Tangshan Sipingtou Chinese landrace; *H*^2^, heritability; $\sigma_{G}^{2}$ and $\sigma_{GE}^{2}$, genotypic variance and genotype-by-environment interaction variance, respectively; ** and ***, significance at *p* < 0.01 and *p* < 0.001, respectively.

**Table 3:** Northern corn leaf blight (NCLB) resistance evaluation results for germplasm groups in Xinzhou2021 and Xinzhou2022.

Group	Number	HR	R	MR	S	HS	Range	Mean
Iodent	32	0	1	4	26	1	2.96–8.29	6.66
Lancaster	49	0	2	25	22	0	3.65–7.34	5.83
LRC	47	0	4	22	20	1	3.73–8.65	5.85
PA	28	0	2	17	9	0	2.88–6.78	5.42
PB	39	0	11	22	6	0	2.70–6.56	4.78
Reid	43	0	0	7	32	4	4.11–8.33	6.66
SPT	39	0	5	17	17	0	3.46–7.15	5.54
Tropical	309	19	204	72	14	0	1.48–7.50	3.51

Note: LRC, Lvda Red Cob; PA and PB, groups A and B germplasms derived from modern US hybrids; SPT, derivatives from the Tangshan Sipingtou Chinese landrace. HR, highly resistant; R, resistant; MR, moderately resistant; S, susceptible; HS, highly susceptible.

**Table 4:** Rating scale for northern corn leaf blight (NCLB) severity and resistance.

Resistance Rating Scores	Description of Leaf Disease	Resistance
1	The area of the leaf lesions is less than 5% of the leaf area.	Highly resistant (HR) (1 ≤ NCLBS_M_ < 2)
3	There are a small number of lesions on the spike leaves, accounting for 6–10% of the leaf area.	Resistant (R) (2 ≤ NCLBS_M_ < 4)
5	There are numerous lesions on the leaves, accounting for 11–30% of the leaf area.	Moderately resistant (MR) (4 ≤ NCLBS_M_ < 6)
7	There are a large number of lesions on the leaves above the ear position, and the lesions are connected, accounting for 31–70% of the leaf area.	Susceptible (S) (6 ≤ NCLBS_M_ < 8)
9	Almost all the leaves of the whole plant are covered by lesions, and the leaves are withering and dying.	Highly susceptible (HS) (8 ≤ NCLBS_M_ ≤ 9)

## Data Availability

All data are contained within the article/supplementary materials.

## Supplementary Materials

The following supporting information can be downloaded at: https://www.mdpi.com/article/10.3390/plants14203171/s1. Table S1: Physiological races, number of planting replicates, and correlation coefficients (*r*) between replicates across the four location-year environment combinations. Table S2: Heterotic group classification of the inbred lines used for northern corn leaf blight (NCLB) evaluation and their mean NCLB scores across four location-year environment combinations. LRC, Lvda Red Cob; PA and PB, groups A and B germplasms derived from modern US hybrids; SPT, derivatives from the Tangshan Sipingtou Chinese landrace. HR, highly resistant; R, resistant; MR, moderately resistant; S, susceptible; HS, highly susceptible. Table S3: Environmental information for the four location-year environment combinations used in the evaluation of northern corn leaf blight (NCLB) resistance.

## Abbreviations

The following abbreviations are used in this manuscript:

Abbreviation	Full term
NCLB	Northern corn leaf blight
QTL	Quantitative trait loci
SS	Stiff Stalk
NSS	Non-Stiff Stalk
ANOVA	Analysis of variance
SPT	Derivatives from the Tangshan Sipingtou Chinese landrace
PA	Group A germplasms derived from modern US hybrids
PB	Group B germplasms derived from modern US hybrids
LRC	Lvda Red Cob
