# Supplementary material for: Identification and Analysis of Resistance to Northern Corn Leaf Blight in Maize Germplasm Resources
Source: Plants (Basel). 2025 Oct 15;14(20):3171. doi: 10.3390/plants14203171 (PMC12566991; doi:10.3390/plants14203171)
Supplement: Supplementary file 1 [file plants-14-03171-s001.zip › Supplementary Table 1.pdf]

Supplementary Table S1 Physiological races, number of planting replicates, and correlation coefficients (*r*) between replicates across the four location-year combinations.

Environments	Races	Number of Replicates	Correlation Coefficient (*r*)
Shangluo 2014	123N	2	0.88
Shangluo 2015	123N	2	0.75
Xinzhou 2021	1, 2, 3, N	4	0.67-0.79
Xinzhou 2022	123N	8	0.74-0.78
